# Betamethasone administration during pregnancy is associated with placental epigenetic changes with implications for inflammation

**DOI:** 10.1186/s13148-021-01153-y

**Published:** 2021-08-26

**Authors:** Darina Czamara, Linda Dieckmann, Simone Röh, Sarah Kraemer, Rebecca C. Rancourt, Sara Sammallahti, Eero Kajantie, Hannele Laivuori, Johan G. Eriksson, Katri Räikkönen, Wolfgang Henrich, Andreas Plagemann, Elisabeth B. Binder, Thorsten Braun, Sonja Entringer

**Affiliations:** 1 Department of Translational Research in Psychiatry, Max-Planck-Institute of Psychiatry, 80804 Munich, Germany; 2 International Max Planck Research School for Translational Psychiatry, München, Germany; 3 Institute of Medical Psychology, Charité − Universitätsmedizin Berlin, Corporate Member of Freie Universität Berlin, Humboldt-Universität Zu Berlin, and Berlin Institute of Health (BIH), Luisenstr. 57, 10117 Berlin, Germany; 4 Department of Experimental Obstetrics, Charité − Universitätsmedizin Berlin, Corporate Member of Freie Universität Berlin, Humboldt-Universität Zu Berlin, and Berlin Institute of Health (BIH), Augustenburger Platz 1, 13353 Berlin, Germany; 5 Department of Child and Adolescent Psychiatry, Erasmus MC, Sophia Children’s Hospital, Rotterdam, The Netherlands; 6 Finnish Institute for Health and Welfare, Helsinki, Finland; 7 Children’s Hospital, Helsinki University Hospital and University of Helsinki, Helsinki, Finland; 8 Faculty of Medicine, PEDEGO Research Unit, MRC Oulu, Oulu University Hospital and University of Oulu, Oulu, Finland; 9 Department of Clinical and Molecular Medicine, Norwegian University of Science and Technology, Trondheim, Norway; 10 Institute for Molecular Medicine Finland, HiLIFE, University of Helsinki, Helsinki, Finland; 11 Medical and Clinical Genetics, University of Helsinki and Helsinki University Hospital, Helsinki, Finland; 12 Department of Obstetrics and Gynecology, Tampere University Hospital and Tampere University, Faculty of Medicine and Health Technology, Tampere, Finland; 13 Department of General Practice and Primary Care, University of Helsinki, Helsinki, Finland; 14 Folkhälsan Research Center, Helsinki, Finland; 15 Department of Obstetrics and Gynaecology and Human Potential Translational Research Programme, Yong Loo Lin School of Medicine, National University of Singapore, Singapore, Singapore; 16 Singapore Institute for Clinical Sciences, Agency for Science, Technology and Research (A*STAR), Singapore, Singapore; 17 Department of Psychology and Logopedics, Faculty of Medicine, University of Helsinki, Helsinki, Finland; 18 Department of Obstetrics, Charité − Universitätsmedizin Berlin, Corporate Member of Freie Universität Berlin, Humboldt-Universität Zu Berlin, and Berlin Institute of Health (BIH), Augustenburger Platz 1, 13353 Berlin, Germany; 19 Department of Psychiatry and Behavioral Sciences, Emory University School of Medicine, Atlanta, GA 30329 USA; 20 Development, Health, and Disease Research Program, University of California, Irvine, Orange, CA USA

**Keywords:** Placenta, DNA methylation, Gene expression, Targeted bisulfite sequencing, *FKBP5*, Betamethasone

## Abstract

**Background:**

Glucocorticoids (GCs) play a pivotal role in fetal programming. Antenatal treatment with synthetic GCs (sGCs) in individuals in danger of preterm labor is common practice. Adverse short- and long-term effects of antenatal sGCs have been reported, but their effects on placental epigenetic characteristics have never been systematically studied in humans.

**Results:**

We tested the association between exposure to the sGC betamethasone (BET) and placental DNA methylation (DNAm) in 52 exposed cases and 84 gestational-age-matched controls. We fine-mapped associated loci using targeted bisulfite sequencing. The association of placental DNAm with gene expression and co-expression analysis on implicated genes was performed in an independent cohort including 494 placentas. Exposure to BET was significantly associated with lower placenta DNAm at an enhancer of *FKBP5*. *FKBP5* (FK506-binding protein 51) is a co-chaperone that modulates glucocorticoid receptor activity. Lower DNAm at this enhancer site was associated with higher expression of *FKBP5* and a co-expressed gene module. This module is enriched for genes associated with preeclampsia and involved in inflammation and immune response.

**Conclusions:**

Our findings suggest that BET exposure during pregnancy associates with few but lasting changes in placental DNAm and may promote a gene expression profile associated with placental dysfunction and increased inflammation. This may represent a pathway mediating GC-associated negative long-term consequences and health outcomes in offspring.

**Supplementary Information:**

The online version contains supplementary material available at 10.1186/s13148-021-01153-y.

## Background

Epidemiological, clinical and experimental studies in animals and humans suggest that suboptimal conditions during intrauterine life impact fetal development as well as a diverse range of physical and mental health outcomes in later life [1]. Glucocorticoids (GCs) play a fundamental role in this fetal “programming” [2]. They are essential for fetal maturation and organ development, and they serve as a common biomarker and mediator across multiple adverse prenatal conditions known to increase risk for offspring psychiatric and somatic disorders [2]. In fact, excess fetal exposure to GCs can permanently alter organ structure and function, predisposing the individual to disease in later life [3]. Results from animal and human studies suggest short- and long-term effects of GC exposure during pregnancy on offspring hypothalamus–pituitary–adrenal-axis function and behavior, as well as on developmental, cognitive, and disease-risk-related outcomes (for reviews see [4–8]). The brain seems to be a particularly sensitive target for the programming effects of prenatal GC exposure. In the fetal brain, GCs play a role in multiple aspects of development, including neurogenesis, gliogenesis, synaptogenesis, and growth of axons and dendrites [9]. Several studies in humans have reported associations between cortisol concentrations during pregnancy and different aspects of offspring brain anatomy and function, as well as on infant and child behavioral outcomes [10–13].

In addition to the effects of endogenous GC during pregnancy, the consequences of prenatal exposure to synthetic GCs (sGC) are of great interest. Since GCs play a vital role in maturation of fetal organ systems, pregnant individuals at risk of preterm labor (currently about 10% of pregnancies) are administered sGC (betamethasone or dexamethasone) that readily pass the placenta to promote fetal lung development and reduce the risk of neonatal morbidity and mortality [14]. Antenatal treatment with sGC in individuals in danger of preterm labor is one of the most cost-effective ways to improve the prognosis of very preterm infants and as such is included in several guidelines as standard prevention [15–17], generally up to 34 or 35 weeks of gestation. Many individuals who receive the treatment go on to deliver at full term [18], but there has also been discussion on expanding the treatment up to 37-week gestation [19], or even up to 39 weeks’ with an elective Cesarean section [20], which would result in several-fold increases in the treatments. However, adverse short- and long-term effects of antenatal treatment with sGC have been reported, including dose-dependent effects on fetal growth and development [21, 22]. Using data from nationwide registries in Finland on more than 670,000 children, a recent paper reports significant associations between sGC treatment during pregnancy and a significant increase in diagnosed childhood mental and behavioral disorders [18].

There are no direct vascular or neural connections between the pregnant person’s and fetal compartments; all exchange and communication are mediated by biological processes that interface with the placenta. Throughout gestation, the placenta receives and transduces signals to and from the pregnant person’s and fetal compartments [23]. Since the placenta is the conduit between the pregnant person’s and fetal environments, it is likely that its function plays a key role in mediating effects of fetal exposures on fetal development and long-term disease risk [4]. The impairment of placental organogenesis and function can have an impact on fetal development, conferring lasting effects on the brain [24, 25]. A study in mice showed that many of the genes regulating placental development are also involved in brain development, specifically of the hypothalamus [26].

Alterations in placental and fetal epigenetic characteristics are thus proposed as a mechanism of long-term biological embedding of prenatal environmental exposures including GCs [5]. Epigenetic alterations within the placenta and their functional consequences have the potential for altering fetal nutrient supply and development [23]. The placenta actively responds to endocrine, immune, and metabolic cues via altered DNA methylation (DNAm) patterns that, in turn are associated with infant neurobehavioral outcome (reviewed in [23]). In fact, sGC have repeatedly been shown to induce both transient as well as lasting changes in DNAm of a number of different tissues. The glucocorticoid receptors (GRs) are nuclear receptors that, once activated by the ligand, bind to specific elements in the DNA, so-called glucocorticoids response elements (GRE). These serve as regulatory elements in a large number of genes to modulate gene expression in a cell-type-specific manner. GCs have been shown to reduce DNAm specifically in these GREs [27, 28] and have been reported to associate with lasting changes in DNAm in these elements, especially bivalent and poised enhancers [29]. Furthermore, GC-induced changes in DNAm in these regions have been associated with changes in gene expression [27–29].

While the effects of GC exposure on DNAm have been investigated in immune and neuronal cells, their effects during pregnancy on placental epigenetic characteristics have, to the best of our knowledge, never been studied systematically in humans. In the current study, we investigated the effects of betamethasone treatment (BET), a sGC with high affinity to the GR, during routine clinical obstetric care (in a “natural experiment” setting) on the DNAm profiles of the placenta at birth [30]. We assessed whether BET leaves epigenetic signatures in the placental epigenome and tested the association between BET exposure and placental DNAm in 136 placental samples (52 with exposure to BET, and 84 gestational-age-matched controls). Afterward, we fine-mapped loci which were associated with BET on an epigenome-wide level with targeted bisulfite sequencing. Furthermore, we checked whether these hits were associated with genetic variants acting as methylation quantitative trait loci (meQTLs) and evaluated possible functional consequences using expression quantitative trait (eQTM) analysis as well as functional annotations of gene networks.

## Results

We assessed the association between placental DNAm levels and several predictor variables, BET in particular, in 136 placental samples. Fifty-two of these samples had been exposed to BET during pregnancy while 84 had not. Sample characteristics are provided in Table 1.

**Table 1:** Characteristics of the study samples

	Betamethasone administration group (BET group, *n* = 52)	Control group (*n* = 84)	*p* value^a^
Mean age of pregnant person in years (SD)	28.46 (6.24)	29.60 (5.51)	0.28
Ethnicity non-European (%)	3 (5.8%)	7 (8.3%)	1.00
Smoking during pregnancy (% yes)	14 (26.92%)	18 (21.42%)	0.60
Mean BMI before pregnancy (SD)	24.30 (5.17)	23.81 (4.21)	0.58
Mean time between BET administration and birth in days (SD)	67.38 (29.87) up to 7 days: 1 7–28 days: 4 28–56 days: 14 over 56 days: 33	NA	NA
Mean gestational age at birth in weeks (SD)	38.03 (2.10)	38.23 (1.88)	0.56
Preterm birth (before week 37)	17 (32.69%)	17 (20.42%)	0.15
Delivery mode: C-section	11 (21.15%)	24 (28.57%)	0.44
Labor	49 (94.23%)	72 (85.71%)	0.21
Parity	1.65 (1.02)	1.97 (1.41)	0.13
Birth weight in grams	3069 (539)	3198 (436)	0.15
Sex (% male)	24 (46.15%)	46 (54.76%)	0.42

The table refers to samples which provide information on phenotypes and methylation levels

*SD* standard deviation

^a^Nominal *p* value from Chi-square test (categorical variables) or t test (quantitative variables)

### Placental DNAm is associated with BET during pregnancy

**Fig. 1:**
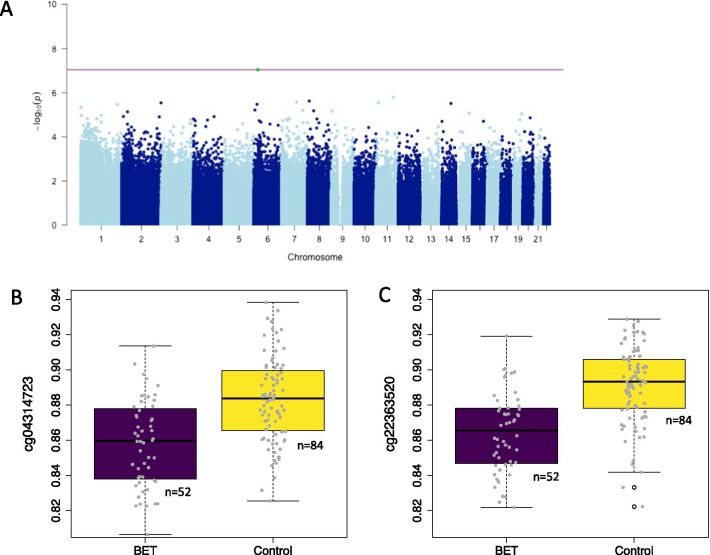
Association results of DNAm levels in the EPIC analysis with betamethasone exposure. Manhattan plot for association of betamethasone exposure (BET) and DNA-methylation level. The position of the CpG-site is depicted on the *x*-axis, the − log10(*p* value) on the y-axis. The red line indicates epigenome-wide significance (**a**). Boxplot for cg22363520 and BET. The *x*-axis denotes the betamethasone exposure group, depicted in purple, and the control group, depicted in yellow. The *y*-axis denotes the methylation levels (**b**). Boxplot for cg04314723 and BET. The *x*-axis denotes the betamethasone exposure group, depicted in purple, and the control group, depicted in yellow. The *y*-axis denotes the methylation levels (**c**)

An overview of all results from the epigenetic analyses is provided in Table 2. We tested the association between BET and placental DNAm levels while adjusting for sex, gestational age, ethnicity, age of pregnant person, smoking during pregnancy and estimated cell type proportions. Ethnicity was determined based on a multi-dimensional scaling (MDS) analysis on the genotypes which yields main axes of genetic variation. The first four axes of variation were used as covariates in the association analysis. Within the group that was exposed to BET, time between BET administration and birth had no significant effect on placental DNAm. Two CpG sites, cg22363520 located in *FKBP5* and cg04314723 located in *LRRC16A*, were significantly methylated between individuals exposed to BET and controls at false discovery rate (FDR) level of 5% (cg22363520 *p* = 4.04 × 10^–11^, cg04314723 *p* = 9.13 × 10^–08^, see Fig. 1a and Additional file 1: Table S1). The association with BET and DNAm of CpG cg22363520 in *FKBP5* remained epigenome-wide significant according to the threshold of Mansell et al. [31], i.e., *p* < 9 × 10^–08^, after further correction for delivery mode, labor and parity (see Table 2, *p* = 1.80 × 10^–10^). Both CpGs showed lower methylation levels in the BET group as compared to the control group (see Fig. 1b, c; mean methylation difference cg22363520: 2.7%, cg04314723: 2.5%). These effect sizes were comparable to the magnitude of methylation differences we found based on child’s sex and gestational age (presented in Additional files 2–5). Sex-stratified analysis indicated no differences in the direction of the BET effect between males and females.

**Table 2:** Overview of epigenetic analyses and results

Methylation data	Research question	Statistical model^a^	Results	Significance threshold
EPIC array	Are CpG-sites on the EPIC array differentially methylated with regard to BET exposure?	summary (lm (beta ~ child’s sex + gestational age + PC1 + PC2 + PC3 + PC4 + pregnant person’s age + smoking during pregnancy + cell type PC1 + cell type PC2 + BET))	**cg22363520** (chr6, bp 35,558,488) is **differentially methylated with BET**	epigenome-wide, *p* < 9 × 10^–08^, based on Mansell et al. [31]
TBS *FKBP5*	Are CpG-sites within *FKBP5* differentially methylated with regard to BET exposure?	summary (lm (beta ~ child’s sex + gestational age + PC1 + PC2 + PC3 + PC4 + pregnant person’s age + smoking during pregnancy + cell type PC1 + cell type PC2 + plate + BET))	**PCR_2_29** (intron7, bp 35,558,488) is **differentially methylated with BET**	FDR 5% over all investigated CpG-sites in *FKBP5*
TBS *FKBP5*	Are CpG-sites within *FKBP5* differentially methylated with regard to the functional variant rs1360780?	summary (lm (beta ~ child’s sex + gestational age + PC1 + PC2 + PC3 + PC4 + pregnant person’s age + smoking during pregnancy + cell type PC1 + cell type PC2 + plate + rs1360780))	**PCR_2_262** (intron 7, bp 35,558,721) **PCR_12_47** (intron 1, bp 35,630,996) **PCR_12_62** (intron 1, bp 35,631,011) **PCR_12_211** (intron 1, bp 35,631,160) **PCR_17_63** (proximal enhancer, bp 35,695,192) **PCR_20_191** (proximal enhancer, bp 35,696,886) are **differentially methylated with rs1360780**	FDR 5% over all investigated CpG-sites in *FKBP5*
TBS *FKBP5*	Are additive effects of the functional variant rs1360780 and BET on DNAm in *FKBP5* present?	summary (lm (beta ~ child’s sex + gestational age + PC1 + PC2 + PC3 + PC4 + pregnant person’s age + smoking during pregnancy + cell type PC1 + cell type PC2 + plate + rs1360780 + BET))	**additive effects of rs1360780 and BET** on DNAm of **PCR_2_29** (intron7, bp 35,558,488)	FDR 5% over all investigated CpG-sites in *FKBP5*
TBS *FKBP5*	Are interactive effects of the functional variant rs1360780 and BET on DNAm in *FKBP5* present?	summary (lm (beta ~ child’s sex + gestational age + PC1 + PC2 + PC3 + PC4 + pregnant person’s age + smoking during pregnancy + cell type PC1 + cell type PC2 + plate + rs1360780 + BET + rs1360780 x BET))	**no interactive effects** after correction for multiple testing	FDR 5% over all investigated CpG-sites in *FKBP5*

PC1–PC4 refer to the first four MDS components, correcting for ethnicity. Cell type PC1, cell type PC2 refer to the first two components from a PCA on the cell types (see Methods). Plate refers to TBS plate (see Methods). bp is the base-pair position (hg19).

^a^Statistical model refers to linear model as set up in R

### Fine mapping of association with BET and placental DNAm in FKBP5 using targeted bisulfite sequencing

The top hit from the epigenome-wide association analysis on BET is located in *FKBP5*, a gene that has been thoroughly studied and identified as a strongly GC responsive gene with many downstream effects via direct protein/protein interaction, including the GR itself (for review [32, 33]). Only a few CpG-sites located in enhancers within *FKBP5* are covered by the EPIC array. We therefore ran targeted bisulfite sequencing (TBS) to fine map methylation levels within functional enhancers including known GREs at the *FKBP5* locus as established by [34].

**Fig. 2:**
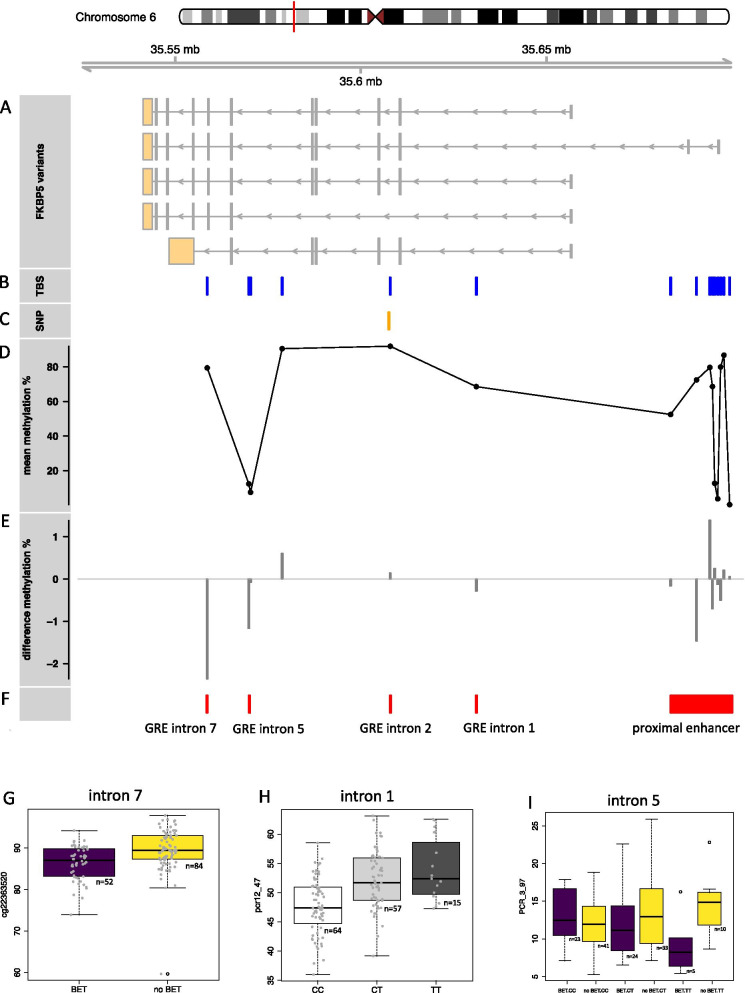
Genomic region of TBS in *FKBP5* and methylation structure. Different transcripts of *FKBP5* (**a**). Genomic positions of TBS amplicons (**b**) and *FKBP5* SNP rs1360780 (**c**). Mean methylation level of all samples across *FKBP5* amplicons (**d**). Mean difference of methylation level between groups per amplicon. A negative value indicates that individuals administered with betamethasone (BET) present with lower methylation levels than control subjects (**e**). Position of functional regions (**f**). Boxplot for association of methylation with BET. The x-axis denotes the BET exposure group, depicted in purple, and the control group, depicted in yellow. The y-axis denotes methylation level in the intronic glucocorticoid response elements (GRE) of intron 7 (**g**). Boxplot for association of rs1360780 and methylation. The x-axis denotes the genotype. The y-axis denotes the methylation level at PCR_12_47 in the intronic GRE of intron 1 (**h**). Boxplot for the interaction of rs1360780 and BET on methylation. The x-axis denotes the combination of genotype and BET, e.g. ‘BET.CC’ indicates the group presenting with BET and a CC genotype while ‘no BET.CT’ indicates the group with no BET exposure and the CT genotype. The *y*-axis denotes methylation level at PCR_3_in the intronic GRE of intron 5 (**i**)

The TBS analysis was performed on the same samples that had been used for the epigenome-wide analysis on the EPIC array. Using this approach, we were able to successfully measure methylation levels at 106 CpG-sites within the *FKBP5* locus (see Fig. 2b). We ran models for association of DNAm of individual CpG-sites with BET exposure. All associations were corrected for multiple testing over all investigated CpG-sites using the Benjamini–Hochberg approach and setting the FDR to 5%. One CpG-site in intron 7 (PCR_2_29), representing the same site as cg22363520, the top hit that had been identified in the epigenome-wide analysis, was associated with BET at FDR-level of 0.05 over all investigated CpG-sites (*p* = 4.11 × 10^–04^, see Fig. 2g, Table 2 and Additional file 6: Table S4). Hence, the association result in *FKBP5* from the EPIC array could be replicated with a different technique. Nine more CpG-sites in intron 5, intron 7 and the proximal enhancer were nominal significantly associated with BET (see Additional file 6: Table S4). Interestingly, the effect of BET on DNAm of 8 of these 10 CpGs was in the same direction as the effect observed for cg22363520 in the epigenome-wide analysis—lower methylation in individuals exposed to BET as compared to controls. The finding that 8 of the 10 identified CpGs show the same direction of effect is more significant as expected by chance (*p* = 0.043, based on the binomial distribution).

### Association of DNAm levels in FKBP5 with genetic variation

Within *FKBP5*, the functional SNP rs1360780 moderates the association between GC exposure and DNAm levels in blood [27]. Specifically, samples presenting with the TT genotype and exposure to dexamethasone showed lower methylation levels of *FKBP5* as compared to samples that had not been exposed to dexamethasone or presenting with a CT/CC genotype. Thus, in the next step we tested if the same genotype also moderated placental DNAm in *FKBP5*. Six CpG-sites in intron 1, in intron 7, and in the proximal enhancers were associated with SNP rs1360780 genotype as main effect at FDR-level of 0.05 over all investigated CpG-sites. The strongest association was found for intron 1 (PCR_12_47, *p* = 1.355 × 10^–08^, see Fig. 2h, Table 2 and Additional file 6: Table S4) where the TT genotype was associated with higher methylation levels. Overall, 13 CpG-sites in intron 1, in intron 7, and in the proximal enhancer were nominal significantly associated with SNP-genotype. While for CpGs located introns 1 and 7, TT was associated with higher methylation levels, effect directions were mixed for CpGs located in the proximal enhancer (see Additional file 6: Table S4). Since BET as well as the rs1360780 genotype were associated with placental DNAm in *FKBP5*, we next checked for combined effects. As effects of SNP-genotype on DNAm were more pronounced as compared to effects of BET, we used the model including genotype as initial model and checked if adding BET to the model significantly increased the model performance. This was true for cg22363520 (PCR_2_29), the CpG that was also associated with BET itself (see Table 2 and Additional file 6: Table S4), here we observed an additive effect of BET and SNP genotype on DNAm. BET as well as CC/CT genotype were associated with lower methylation levels (see Additional file 7: Fig. S2). We found no significant interaction effects between SNP and BET for cg22363520 or any other CpG-site after multiple testing correction (see Table 2). Ten CpG-sites in intron 1, in intron 5, and in the proximal enhancer presented with nominal significant interaction p-values. The strongest effect was observed in intron 5 (PCR_3_97, *p* = 1.22 × 10^–02^, Fig. 2i and Additional file 6: Table S4) with individuals with TT and BET showing lower methylation levels. However, it is has to be noted that this group only consisted of five individuals.

### Association between placental FKBP5 methylation and placenta FKBP5 gene expression in the intrauterine sampling in early pregnancy study (ITU cohort)

Our results indicate that BET exposure is associated with placental demethylation of *FKBP5*. Several other studies have shown that demethylation of this enhancer in *FKBP5* is associated with higher *FKBP5* RNA expression [28, 35]. As no RNA expression levels were available for our initial cohort, we used additional data from the intrauterine sampling in early pregnancy study (ITU cohort, see Methods), a prospective, longitudinal pregnancy cohort study with placental gene expression available for 494 samples. Demographics of the ITU and differences to our initial cohort are given in Table 3.

**Table 3:** Demographics of ITU study

Phenotype	ITU study (*n* = 494)	BET study (*n* = 136)	*p *value
Mean age of pregnant person in years (SD)	34.62 (4.82)	29.16 (5.80)	**< 0.01**
BET exposure	12 (2.43%)	52 (38.24%)	**< 0.01**
Smoking during pregnancy (% yes)	12 (2.43%)	32 (23.53%)	**< 0.01**
Mean BMI before pregnancy (SD)	23.82 (4.14)	23.41 (3.68)	0.50
Mean gestational age in weeks (SD)	40.01 (1.61)	38.16 (1.96)	**< 0.01**
Premature birth (before week 37)	18 (3.64%)	34 (25%)	**< 0.01**
Birth weight in grams	3533.82 (503.17)	3148.79 (480.09)	** < 0.01**
Sex (% male)	252 (51.01%)	70 (51.47%)	1.00

*SD* standard deviation

*p* value: nominal *p* value from Chi-square test (categorical variables) or *t *test (quantitative variables) comparing the initial BET study to the ITU study. Nominally significant *p* values are depicted in bold

**Fig. 3:**
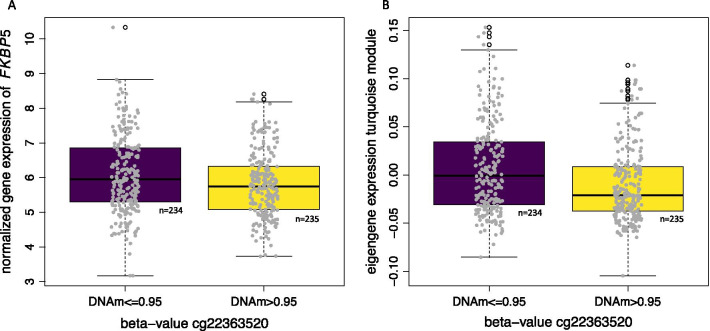
Boxplot of placental DNAm and gene expression levels. The *x*-axis denotes placental DNAm of cg22363520, based on median split (beta-value of 0.9522), beta-values below or equal to the median are depicted in purple, beta-values above the median depicted in yellow. The y-axis denotes gene expression levels of FKBP5 after variance-stabilizing transformation. The association stays significant after exclusion of one potential outlier in the group below the median (*p* = 0.0036) (**a**). The *x*-axis denotes placental DNAm of cg22363520, based on median split (beta-value of 0.9522), beta-values below or equal to the median are depicted in purple, beta-values above the median depicted in yellow. The *y*-axis denotes the eigengene of the turquoise module (**b**)

Using data from the ITU study, we could replicate previous findings also in placental tissue: lower methylation levels at cg22363520 were associated with increased expression of *FKBP5* in placentas sampled at birth (*p* = 0.003, correlation *r* = − 0.28, see Fig. 3a), hence cg22363520 is an expression quantitative trait methylation locus (eQTM). This association remained stable after correction for delivery mode, labor, parity, maternal hypertension and maternal diabetes (*p* = 0.004). In the ITU cohort, there were only 12 samples with prenatal BET exposure, therefore power was too low to replicate the association between BET and cg22363520.

### Weighted gene co-expression network analysis (WGCNA) in placenta in the ITU cohort

Having supportive evidence for the effect of BET on *FKBP5* methylation and the correlation of low methylation with higher *FKBP5* mRNA expression in placenta, we wanted to further explore co-expression of other genes with *FKBP5* in the placenta. For this purpose, we calculated weighted gene co-expression networks (WGCNA) Based on pair-wise correlations, this method defines clusters, so called modules, of correlated genes. From the ITU cohort, 494 placental samples with 3’prime RNA sequencing were available. After pre-processing, as described in Additional file 13, variance-stabilizing transformation to the raw counts of 8245 transcripts from 494 individuals was performed and used as input for WGCNA. Overall, we found 17 gene modules of highly co-expressed genes ranging in size from 43 up to 1727 genes. *FKBP5* was assigned to the largest module, the turquoise module (*n* = 1727 genes, see Additional file 8: Table S5). As we observed that lower methylation at cg22363520 was associated with higher gene expression of *FKBP5*, we also checked for correlation of the other genes in the turquoise module*.* Gene expression of 677 genes in the turquoise module was significantly correlated with methylation levels of cg22363520 at FDR of 5%. The majority of these genes (*n* = 655 genes) showed the same effect direction as *FKBP5*, i.e., lower methylation at cg22363520 was associated with higher gene expression (see Additional file 8: Table S5). The same direction of effect was present when we looked into the eigengene, i.e., the first principal component of the standardized expression profiles of the 1727 genes of the turquoise module (*r* = − 0.33, *p* = 1.702 × 10^–13^, see Fig. 3b). The turquoise module was not associated with delivery mode, labor, parity, maternal diabetes or maternal hypertensive disorders.

Next, we evaluated whether genes in the turquoise module had previously been associated with prenatal stress exposure in general. Interestingly, the turquoise module contains genes for which placental gene expression differences have been reported with regard to exposure to prenatal depression and anxiety in humans or physiological stress in mice and rats ([36–40], see Additional file 9: Table S6). Furthermore, the gene meta-signature of the pre-eclamptic placental transcriptome established in van Uitert et al. [41] overlapped with genes in the turquoise module (see Additional file 10: Table S7). Of the 388 genes identified in Uitert et al., 323 were also available in the ITU dataset after preprocessing and 60 of these overlapped with genes from the turquoise module. To establish whether this overlap was significant, we used 1000 random gene subsets. For each subset, we randomly picked 323 genes out of the 8245 overall transcripts available in the ITU cohort and evaluated how many genes in the random subset overlapped with genes from the turquoise module. In none of these random subsets, the overlap was larger than 60, in fact all random subsets presented with overlaps below 25. This indicates that the overlap of 60, which we found for the original dataset, was higher as expected by chance and hence significant (empirical *p* value = 1/1001 = 9.99 × 10^–04^).

While the turquoise module itself was not associated with pre-eclampsia or with hypertensive pregnancy disorders in general in the ITU cohort (*n* = 30 samples with hypertensive pregnancy disorders), the majority of genes overlapping with genes identified in Uitert et al. [41] showed the same effect direction in both studies: positive correlation with *FKBP5* gene expression (which was associated with BET) as well as differential gene expression with higher gene expression in pre-eclampsia as reported in [41].

**Fig. 4:**
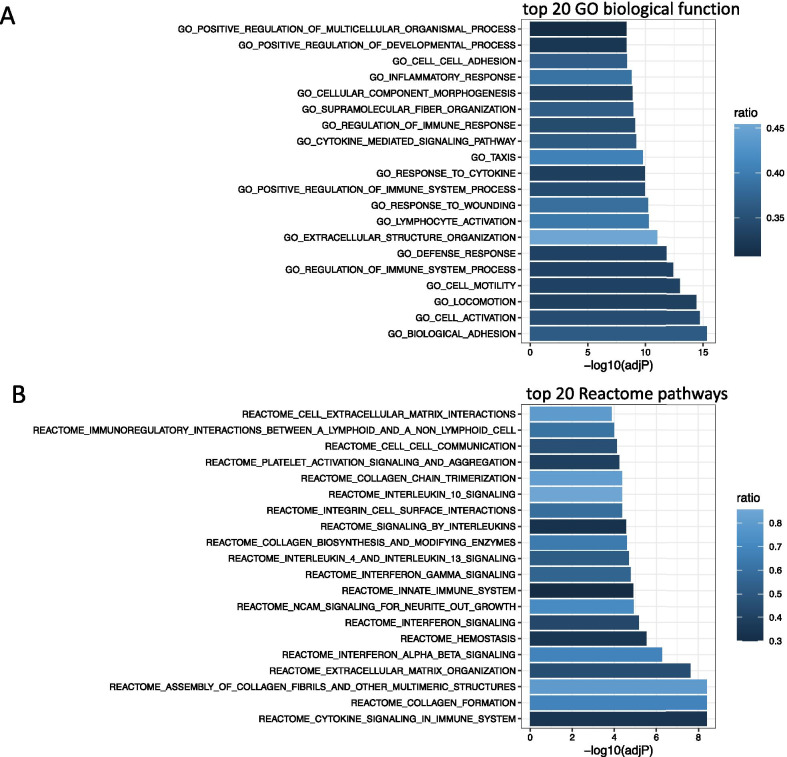
Enrichment analysis for turquoise gene module. The *p* values are depicted on the *x*-axis, the specific term which was tested for enrichment on the *y*-axis. Ratio denotes the ratio between overlapping genes and all genes in the respective pathway. Top 20 results for enrichment with GO biological function pathways (**a**). Top 20 results for enrichment with reactome pathways (**b**)

The turquoise module was highly enriched for a variety of GO and Reactome terms (see Additional file 11: Table S8 and Additional file 12: Table S9). The top 20 terms including pathways involved in inflammation (such as “inflammatory responses”, “signaling by interleukins”, “cytokine signaling in immune system”) and immune response (“regulation of immune response”, “regulation of immune system process”, “innate immune system”, see Fig. 4).

## Discussion

While GCs are crucial for fetal maturation and organ development, excess fetal exposure to GCs has been associated with higher disease risk in later life. In fact, significant associations between sGC treatment during pregnancy and diagnosed childhood mental and behavioral disorders have been reported [18]. The placenta plays a key role in mediating effects of fetal exposures and thus changes in placental epigenetic signatures could provide a possible mechanism of long-term biological embedding of prenatal environmental exposures including GCs [5]. However, effects of GC exposure during pregnancy on placental epigenetic signatures in humans have not been systematically explored.

In this study, we investigated the association of BET during pregnancy on changes in epigenetic placental signatures. We compared 52 placental samples of individuals who had been treated with BET during pregnancy to 84 gestational-age-matched placental control samples. An epigenome-wide association analysis revealed one CpG site, cg22363520 located in *FKBP5*, with statistical significance on an epigenome-wide level (*p* < 9.80 × 10^–08^ [42]). We confirmed this finding with a different technique and fine-mapped the *FKBP5* locus using targeted bisulfite sequencing.On average, BET cases presented with 2.7% lower DNAm levels at cg22363520, as compared to controls (see Fig. 1b). This is comparable to the effect we observed for gestational age (on average 2.5% methylation difference, 59 significant CpG sites) and a bit lower as compared to effect sizes for sex-specific differences (on average 3.5% methylation difference, 2234 significant CpG sites). Exposure to BET took place on average about two months before birth when placental DNAm levels were assessed. Given the half-life of BET, no direct pharmacological effects are expected 72 h after treatment [43] while plasma betamethasone can be measured up to 14 days after treatment [44]. Thus, we were only able to observe effects that persist even in the absence of any active drug. We identified just one epigenome-wide significant CpG site but might have identified more if DNAm had been assessed shortly after exposure to BET. With regard to dexamethasone (DEX), it has already been shown that DEX-induced effects on DNAm are mostly dynamic and revert after stopping the exposure, but also some lasting effects have been reported [27, 29].

While we are the first to report BET-induced DNAm changes in the placenta with *FKBP5*, induced DNAm changes in *FKBP5* in association with other stressors have already been observed: Monk et al. [45] reported an association between placental DNAm in *FKBP5* and perceived maternal stress and Paquette et al. [35] observed that placental DNAm in *FKBP5* was associated with children’s neurobehavioral outcomes. CpG cg22363520 is located within an intronic GRE for which GR-agonist induced demethylation has been identified in other tissues, such as immune cells or brain cells [27, 29]. In fact, the *FKBP5* locus contains a number of GREs both intronic as well as in the upstream enhancer regions, and for CpGs sites within the GRE, demethylation has been reported [27]. The extent of demethylation of GRE with GC exposure, however, seems to be tissue dependent [27, 29]. It has been proposed that binding of the activated GR to binding elements leads to reduction in local DNAm via DNA excision/repair mechanisms [46]. Demethylation of these GRE has been shown to associate with enhanced *FKBP5* transcription, specifically after GC stimulation [28].

GC or stress-associated changes in DNAm of *FKBP5* GRE have also been shown to be moderated by a common functional variant, rs1360780 [27]. The T/A allele of rs1360780 is associated with enhanced transcription of *FKBP5* by generating a TATA-Box binding site and looping of an enhancer in intron 2 to the transcription start site. In the context of GR activation, this allele is also associated with enhanced demethylation of intronic GREs (mainly observed for intron 7 GREs), at least in immune cells [28]. Overall, the combination of this allele with stress-exposure is associated with a more responsive *FKBP5* expression to GCs. Our findings suggest that such interaction effects of SNP and GR-agonist are not pronounced strongly in placental tissue, even though main genetic effects are observed for a number of CpG sites (see Fig. 2h and Additional file 6: Table S4). Additive effects of genotype and exposure are, however, possible (see Additional File 7: Fig. S2).

Demethylation of GRE within the *FKBP5* locus has been associated with higher mRNA induction. *FKBP5* encodes a peptidylprolylisomerase, the immunophilin FKBP51, that influences the function of a large number of pathways via direct protein–protein interactions including with several executers of posttranslational modifications in different tissues [47]. Altering the epigenetic setpoint of *FKBP5* regulation, thus likely has far reaching consequences on cell function [48]. In our initial BET cohort, exposure to BET was associated with placental demethylation of *FKBP5* at cg22363520 in intron 7. As *FKBP5* gene expression levels were not available for this cohort, we evaluated possible functional effects of cg22363520 in an independent Finnish cohort (ITU) where 494 placentas with DNAm as well as gene expression at birth were available. In ITU, lower DNAm in cg22363520 correlated with higher *FKBP5* expression (*r* = − 0.27, *p* = 0.003, see Fig. 3a). This is consistent with data from a previous study [35] that found that placenta mean DNAm of *FKBP5* intron 7 was negatively correlated with *FKBP5* mRNA level. While in the same direction and of similar magnitude (*r* = − 0.22, *p* = 0.08), the correlation did not reach statistical significance possibly due to the smaller sample size (*n* = 63).

To further explore the possible consequences of increased *FKBP5* expression with BET exposure, we explored placental co-expression networks. Using WGCNA, we identified 17 co-expressed gene modules, of which the largest with 1727 co-correlated genes, included *FKBP5*. The overall expression of this gene module highly significantly correlated with cg22363520 methylation (*r* = − 0.33, *p* = 1.702 × 10^–13^, see Fig. 3b), suggesting the BET-associated changes in DNAm of this site correlate with gene expression changes of this cluster, with lower methylation associated with higher expression. Interestingly, this module significantly overlaps with genes that have previously been associated with preeclampsia [41], with genes upregulated in preeclampsia also showing positive correlation with *FKBP5* expression. This suggests that transcriptional signatures associated with higher *FKBP5* expression (and lower cg22363520 methylation) could be indicative of placental dysfunction. The gene module was also enriched for inflammatory as well as for immune response pathways. In fact, FKBP51 has been shown to moderate immune pathways [49] via for example direct interaction with regulators of the alternative NFkappaB pathways leading to a proinflammatory immune response [50]. This is of interest, given the fact that long-term effects of BET have been associated with childhood mental and behavioral disorders [18] and that maternal inflammation has indeed been suggested as possible mediator between maternal stress and offspring neuropsychiatric risk [51, 52]. This may extend to placental inflammation. In fact, in rodents, prenatal stress was shown to lead to placental and fetal brain inflammation, including an elevation in the chemokine *Ccl2,* as well as to negative behavioral consequences in offspring [38]. In our data, *CCL2* was among the transcripts of the turquoise module showing higher expression with lower *FKBP5* methylation in the ITU sample and with positive correlation with *FKBP5* mRNA expression (*r* = 0.45, *p* = 1.33 × 10^–25^). In an animal model, knock-out of *Ccl2* prevented prenatal stress induced deficits in sociability and anxiety-like behavior in the offspring [38]. BET-induced changes in placental DNAm may thus mediate negative health outcomes by promoting a pro-inflammatory profile in this tissue.

Finally, strength and limitations of our study should be mentioned. Our initial BET study took place in a clinically well-characterized sample. Furthermore, due to exclusion of pregnancies complicated by diabetes, hypertension, preeclampsia and eclampsia, our initial sample is more homogenous reducing possible confounding effects of other factors. As the BET model has been proven to have fetal and placental programming effects [4, 21, 22], studying epigenetic effects in this framework seems justified. Furthermore, we evaluated epigenetic effects using two different techniques, EPIC arrays as well as targeted bisulfite sequencing. In a second, independent cohort, the ITU cohort, we assessed placental DNAm as well as gene expression on a genome-wide level. Especially for the latter, so far only few studies investigated human placental gene expression on a transcriptome-wide level. On the other hand, our study also presents with limitations. Our initial sample represents a relatively small sample size of 136. Thus, we might have missed CpG sites associated with BET but which would present with small effect sizes. Furthermore, as all individuals in the BET group were exposed to the same dose of 2 × 12 mg, testing of dose–response effects was not possible. Further, we cannot determine whether our findings are related to BET itself or to the clinical situation in which it was administered. We also cannot rule out that differences in placental methylation preceded, rather than were caused by BET, to rule out reverse causation, placenta samples need to be collected before treatment, which in most cases is not feasible. As shown in [18], associations between antenatal corticosteroid treatment and mental and behavioral disorders are more characteristic to children born at term. In our initial cohort BET was associated with lower *FKBP5* methylation also after exclusion of pre-term births (*p* = 7.41 × 10^–08^). However, as only 34 individuals (17 controls and 17 with BET exposure) presented with preterm births, we do not have enough power to look more closely into aggregated effects of pre-term birth and BET. Another limitation is that placental sampling in the initial BET study followed a different protocol based on random sampling from whole placental biopsies, while the ITU study used placental samples from the fetal side. The choice of sampling protocol can influence the outcome [53] and we cannot rule out the possibility that our results might be biased due to the different approaches.

In our initial sample, we did not follow-up of the children and cannot directly test for effect of BET on the child’s health. In future studies, it would be interesting to assess BET exposure during pregnancy, DNAm and gene expression in placenta and cord blood as well as child outcomes. In such an approach, mediation analysis testing if DNAm or gene expression mediate the association between BET and child health could be directly tested for.

## Conclusions

Taken together, our findings indicate that BET exposure is associated with specific changes in placental DNA methylation in enhancers of *FKBP5* that may associate with higher gene expression of *FKPB5.* Pathways analysis suggests that lower DNA methylation at this site and higher gene expression of *FKBP5* may act in a correlated gene module that includes transcripts associated with placental dysfunction and is enriched for genes involved in inflammation and immune response. Maternal inflammation during pregnancy has already been associated with childhood outcomes including neurodevelopmental disorders (for review: [51]), newborn brain connectivity and offspring working memory [54]) and in fact, has been suggested as a possible mediator between maternal stress and offspring neuropsychiatric risk [55]. Our findings suggest that maternal BET exposure could impact placental inflammation as a possible mediator of lasting effects on child’s developmental and health outcomes.

## Methods and materials

### Study population

Pregnant individuals exposed to a single course of antenatal BET (Celestan®, MSD GmbH, Haar, Germany) for fetal lung maturation (2 × 12 mg intramuscular; *n* = 54) between 23 + 5 to 34 + 0 wks (weeks + days of gestation) were recruited prospectively before birth and compared to a gestational-age-matched control group that received no antenatal BET (*n* = 85 [22, 30]). The control group consisted of gestational age (weeks) and sex-matched pregnant women at the time of delivery [22]. Healthy pregnant individuals with singletons were recruited in parallel, with the same exclusion and inclusion criteria as for the treatment group, only without antenatal steroid treatment during pregnancy. Exclusion criteria were: multiple pregnancy, severe fetal malformations, repeated courses of BET or administration of glucocorticoids other than BET, estimated weight below the 5th and exceeding the 95th percentile [56], pregnancies complicated by diabetes, hypertension, preeclampsia and eclampsia. The groups did not differ with regard to pregnant person’s age or BMI, gestational age, parity or delivery mode (see Table 1). This study was approved by the Ethics Committee of the Charité - Universitätsmedizin, Berlin, Germany (EA2-149-07).

### Sampling

All participants were coded, thus blinding their treatment status to experimenters/researchers. Whole placental biopsies were taken as previously described using a systematic and uniform random sampling protocol [22, 57]. Placenta biopsies were collected with an overlaying grid placed on top of the placenta. While the position of the first tissue section was chosen randomly, the other biopsies were selected according to the pre-determined patterns of the grid within the peripheral and central zone of the placenta [22]. Sampling was started immediately after delivery within the labor ward in a special placenta room. Samples were snap frozen in liquid nitrogen and stored at − 80 °C. Human placental samples were homogenized in Lysing Matrix D tubes using the FastPrep 24 (MP Biomedicals). The Quick-DNA™ Universal Kit (Zymo Research, Freiburg, Germany) was used to extract DNA according to the manufacturer's instruction. Concentration of DNA and its degree of purity were checked by a FLUOstar Omega spectrometer (BMG Laptech, Ortenberg, Germany). DNA samples needed to show a minimal purity of A260/A280 ≥ 1.8.

### Genotyping and MDS components

Genotyping was performed using the Illumina Infinium Global Screening Array containing 663,320 SNPs. Only markers with a callrate of at least 98%, a minor allele frequency of at least 1% and a p value for deviation from Hardy–Weinberg Equilibrium > 1.0 × 10^−06^ were kept in the analysis. Furthermore, individuals with a callrate below 98% were removed. All individuals were unrelated (pi-hat, i.e., estimated IBD fraction, < 0.125 with any other sample). The final dataset included 297,956 SNPs and 137 IDs. The quality control-pipeline was set up in PLINK 1.9 [58]. MDS components were calculated on the IBD matrix of the pruned genotype data using PLINK, a window-size of 200, a shift of 100 and a *r*^2^-value of 0.2. The first four MDS components, explaining 60% of genetic variability, were included as covariates in subsequent analyses.

### DNA methylation

Extracted DNA from 139 placenta samples was run on Illumina Infinium EPIC Methylation arrays. The quality control pipeline was set up using the R-package *minfi* 1.36.0 [59]. Two samples were excluded as they showed artefacts in the beta-value distribution. Methylation beta-values were normalized using the swan normalization [60]. After normalization, slide was the most significant batch and removed using the Combat function in the R-package *sva* 3.30.1 [61]. We excluded any probes on chromosome X or Y, probes containing SNPs and cross-hybridizing probes according to [62–64]. Furthermore, any CpGs with a detection *p* value > 0.01 in at least 25% of the samples were excluded. The final dataset contained 793,213 CpGs and 137 participants. For 136 of these (52 BET and 84 controls), genotypes and hence MDS components were available. Using *MixUpMapper* 1.2.4 [65] we confirmed that methylation levels and genotypes matched and that no sample mix-ups were present.

### Targeted bisulfite sequencing of the FKBP5 locus

This method has been described in detail in [34] and is explained in detail in Additional files 13 and 14: Table S10. In brief, 106 CpG sites could be successfully called and were subjected to further analyses.

### Statistical analysis

All statistical analyses were set up in R 3.5.2 (https://www.r-project.org) using beta-values derived from DNAm. We used the R package *Gviz 1.34.1* [66] for plotting of genomic regions and color palettes from the R-package *viridis* 0.6.1 (https://sjmgarnier.github.io/viridis/).

#### Cell type composition

Placental cell type composition was derived using the R-package *planet* 0.99.3 by applying the robust partial correlation algorithm [67] and based on the reference sample described in [68]. While no major differences in estimated cell type proportions of endothelial cells, Hofbauer cells, nucleated red blood cells, syncytiotrophoblasts and trophoblasts between BET cases and the control group were detected, the BET group presented with a slightly higher proportion of stromal cells as compared to the control group (on average 0.9% difference, *p* value = 0.03, see Additional file 15: Table S11). As cell type proportions are highly correlated with each other, we ran a principal component analysis across all six estimated cell types and used the first two principal components, explaining more than 70% of the overall variance in cell type proportions, as covariates.

#### Epigenome-wide association studies (EWAS)

Association of beta methylation levels of EPIC CpG sites and outcomes were computed using linear models and the R-package *CpGassoc* 2.60 [69]. For our primary analysis, EWAS on BET, child’s sex, gestational age, the first four MDS components, pregnant person’s age, smoking during pregnancy and the first two PCs from estimated cell type proportions were included as covariates (see Additional file 13).

#### Targeted bisulfite sequencing (TBS)

We applied a two-step approach: first, we conducted an EWAS using Infinium Methylation EPIC arrays to test the association between BET and placental DNAm patterns. Second, as the EPIC array offers only limited coverage for enhancer regions [70], we fine-mapped genomic regions significantly associated with BET on an epigenome-wide level using TBS. For TBS, individual methylation levels were associated with BET and SNP genotype using linear models. CpG sites were stratified into functional groups, based on their genomic position (GRE intron 1, GRE intron 2, GRE intron 5, GRE intron 7, and proximal enhancer (see Additional file 14: Table S10). These functional regions have been described in [27]. Child’s sex, gestational age, the first four MDS components, pregnant person’s age, smoking during pregnancy as well as the first two principal components of estimated cell types and plate were included as covariates. Plate was included as factorial covariate as we observed that coverage and conversion rates significantly differed between the two plates.

P values were corrected at a false-discovery rate (FDR) cutoff of 5% using the Benjamini–Hochberg approach.

### Sampling and statistical analysis in the ITU cohort

As no gene expression levels are available in our initial cohort, we used the data from an independent cohort, the intrauterine sampling in early pregnancy study (ITU) cohort, for analysis of expression quantitative methylation as well as co-expression network analysis.

#### Sampling

The ITU cohort consists of Finnish pregnant individuals and their children born between 2012–2017. The cohort is described in [71]. ITU is a prospective, longitudinal pregnancy cohort study comprising a total of 944 individuals, 494 of whom had data available on placental gene expression and could thus be included in the current study. As explained in more detail in Additional file 13, pregnant individuals were recruited at maternity clinics and through the Helsinki and Uusimaa Hospital District Fetomaternal Medical Center in Finland. Eligibility criteria included singleton pregnancy, no prenatal diagnosis of chromosomal abnormality, pergnant person’s age ≥ 18 years, and sufficient Finnish language ability to ensure informed consent. As opposed to our initial cohort, the ITU cohort also included individuals with diabetes (*n* = 109) and hypertensive disorders (*n* = 30).

Placenta samples from the fetal side of the placenta, relatively close to the umbilical cord were collected after birth by midwives who took nine-site biopsies within 120 min of delivery. The biopsies were stored in RNA storage solution until research staff collected the samples (within 24 h of delivery), which were stored at − 80 °C.

Demographic information on the ITU cohort is depicted in Table 3. As compared to the BET cohort, ITU participants were older and smoked less during pregnancy. Furthermore, the ITU cohort contained fewer individuals with BET exposure (*n* = 12) and fewer premature births, and children in ITU presented with higher birth weight.

#### RNA sequencing in the ITU cohort

For gene expression analyses DNA from placenta was carried out using a bead-based method optimized by tissue type (Chemagic 360 Perkin Elmer). The QuantSeq 3' mRNA-Seq Library Prep Kit (Lexogen) was used to generate messenger RNA (mRNA) sequencing libraries. All libraries were multiplexed and sequenced on an Illumina HighSeq4000 system at a depth of 10 million reads per mRNA library. Adapter sequences were trimmed using cutadapt (https://cutadapt.readthedocs.io/en/stable/) and sequenced reads were aligned to the human genome reference using the STAR aligner [72]. We performed featureCounts from the R-package *rsubread* 1.22.1 [73]. We filtered the datasets to genes presenting with a raw count of at least 10 in at least 90% of the individuals, resulting in a final dataset including 8245 transcripts and 494 individuals. Surrogate variable (SV) analysis was used to correct for possible batch effects [61], the first SV was detected as significant and used as covariates in the subsequent analyses.

Weighted gene co-expression network and expression quantitative methylation analysis are explained in detail in the Additional file 13.

#### Pathway enrichment analysis

Gene-set enrichments were tested using FUMA’s GENE2FUNC v1.3.5 [74] setting the FDR adjusted p values for enrichment to 0.05 and considering Gene Ontology (GO) terms. We used the list of all genes in the turquoise module (*n* = 1727) as input and all tested genes (*n* = 8245) as background list.

## Supplementary Information

**Additional file 1: Table S1**. EPIC CpGs significantly associated with BET

**Additional file 2**. Supplementary EWAS on child’s sex and gestational age

**Additional file 3: Figure S1**. Association of DNAm levels with child’s sex and gestational age. Manhattan plot for association of child’s sex with DNA-methylation level. The position of the CpG site is depicted on the x-axis, the –log10(*p* value) on the y-axis. The red line indicates epigenome-wide significance, the blue line significance at FDR of 0.05 (A). Manhattan plot for association of gestational age with DNA-methylation level. The position of the CpG-site is depicted on the x-axis, the –log10(p value) on the y-axis. The red line indicates epigenome-wide significance, the blue line significance at FDR of 0.05 (B)

**Additional file 4: Table S2**. EPIC CpGs significantly associated with child’s sex

**Additional file 5: Table S3**. EPIC CpGs significantly associated with gestational age

**Additional file 6: Table S4**. Association results of TBS analysis

**Additional file 7: Figure S2**. Boxplot of placental DNAm at cg22363520 and BET + SNP genotype in the TBS analysis. Boxplot of placental DNAm at cg22363520 and BET. The y-axis denotes placental DNAm of cg22363520, the x-axis denotes the betamethasone exposure group, depicted in purple, and the control group, depicted in yellow (A). Boxplot of placental DNAm at cg22363520 and rs1360780 genotype. The y-axis denotes placental DNAm of cg22363520, the x-axis denotes rs1360780 genotype (B). Boxplot of placental DNAm at cg22363520 and BET+ rs1360780 genotype. The y-axis denotes denotes placental DNAm of cg22363520, the x-axis denotes rs1360780 genotype and BET, e.g. ‘BET.CC‘ indicates the group presenting with BET and a CC genotype while ‘no BET.CT‘ indicates the group with no BET exposure and the CT genotype (C).

**Additional file 8: Table S5**. Co-expressed genes in the turquoise WGCNA module and association with cg22363520

**Additional file 9: Table S6**. Genes in the turquoise WGCNA module associated with prenatal stress

**Additional file 10: Table S7**. Genes from turquoise module overlapping with differential expressed genes in Uitert et al.

**Additional file 11: Table S8**. Biological function GO terms which are significantly enriched for genes from the turquoise module at FDR-level of 5%

**Additional file 12: Table S9**. Reactome terms which are significantly enriched for genes from the turquoise module at FDR-level of 5%

**Additional file 13**. Supplementary Methods

**Additional file 14: Table S10**. Amplicons in TBS

**Additional file 15: Table S11**. Estimated cell type proportions in whole sample, BET and controls

## Declarations

### Availability of data and materials

Due to the sensitive nature of the patient data used in our study and consent, the data sets are not and cannot be made publicly available. An interested researcher can obtain a de-identified data set after approval from the Study Boards. Data requests may be subject to further review by the national register authority and by the ethical committees.

## References

[1] Entringer S, Buss C, Wadhwa PD (2015). Prenatal stress, development, health and disease risk: a psychobiological perspective-2015 Curt Richter Award Paper. Psychoneuroendocrinology.

[2] Cottrell EC, Seckl JR, Holmes MC, Wyrwoll CS (2014). Foetal and placental 11beta-HSD2: a hub for developmental programming. Acta Physiol (Oxf).

[3] Harris A, Seckl J (2011). Glucocorticoids, prenatal stress and the programming of disease. Horm Behav.

[4] Braun T, Challis JR, Newnham JP, Sloboda DM (2013). Early-life glucocorticoid exposure: the hypothalamic-pituitary-adrenal axis, placental function, and long-term disease risk. Endocr Rev.

[5] Krontira AC, Cruceanu C, Binder EB (2020). Glucocorticoids as mediators of adverse outcomes of prenatal stress. Trends Neurosci.

[6] McGowan PO, Matthews SG (2018). Prenatal stress, glucocorticoids, and developmental programming of the stress response. Endocrinology.

[7] Reynolds RM (2013). Glucocorticoid excess and the developmental origins of disease: two decades of testing the hypothesis–2012 Curt Richter Award Winner. Psychoneuroendocrinology.

[8] Wyrwoll CS, Holmes MC, Seckl JR (2011). 11beta-hydroxysteroid dehydrogenases and the brain: from zero to hero, a decade of progress. Front Neuroendocrinol.

[9] Moisiadis VG, Matthews SG (2014). Glucocorticoids and fetal programming part 2: mechanisms. Nat Rev Endocrinol.

[10] Buss C, Davis EP, Shahbaba B, Pruessner JC, Head K, Sandman CA (2012). Maternal cortisol over the course of pregnancy and subsequent child amygdala and hippocampus volumes and affective problems. Proc Natl Acad Sci USA.

[11] Davis EP, Glynn LM, Schetter CD, Hobel C, Chicz-Demet A, Sandman CA (2007). Prenatal exposure to maternal depression and cortisol influences infant temperament. J Am Acad Child Adolesc Psychiatry.

[12] de Weerth C, van Hees Y, Buitelaar JK (2003). Prenatal maternal cortisol levels and infant behavior during the first 5 months. Early Hum Dev.

[13] Graham AM, Rasmussen JM, Entringer S, Ben Ward E, Rudolph MD, Gilmore JH (2019). Maternal cortisol concentrations during pregnancy and sex-specific associations with neonatal amygdala connectivity and emerging internalizing behaviors. Biol Psychiatry.

[14] McGoldrick E, Stewart F, Parker R, Dalziel SR (2020). Antenatal corticosteroids for accelerating fetal lung maturation for women at risk of preterm birth. Cochrane Database Syst Rev.

[15] Committee on Obstetric P. Committee Opinion No. 713: Antenatal Corticosteroid Therapy for Fetal Maturation. Obstet Gynecol. 2017;130(2):e102–e9. 10.1097/AOG.0000000000002237 28742678

[16] Sweet DG, Carnielli V, Greisen G, Hallman M, Ozek E, Te Pas A (2019). European consensus guidelines on the management of respiratory distress syndrome—2019 update. Neonatology.

[17] WHO. WHO Recommendations on Interventions to Improve Preterm Birth Outcomes. WHO Recommendations on Interventions to Improve Preterm Birth Outcomes. WHO Guidelines Approved by the Guidelines Review Committee. Geneva; 2015.

[18] Raikkonen K, Gissler M, Kajantie E (2020). Associations between maternal antenatal corticosteroid treatment and mental and behavioral disorders in children. JAMA.

[19] Gyamfi-Bannerman C, Thom EA, Blackwell SC, Tita AT, Reddy UM, Saade GR (2016). Antenatal betamethasone for women at risk for late preterm delivery. N Engl J Med.

[20] Sotiriadis A, Makrydimas G, Papatheodorou S, Ioannidis JP, McGoldrick E (2018). Corticosteroids for preventing neonatal respiratory morbidity after elective caesarean section at term. Cochrane Database Syst Rev.

[21] Braun T, Sloboda DM, Tutschek B, Harder T, Challis JR, Dudenhausen JW (2015). Fetal and neonatal outcomes after term and preterm delivery following betamethasone administration. Int J Gynaecol Obstet.

[22] Braun T, Husar A, Challis JR, Dudenhausen JW, Henrich W, Plagemann A (2013). Growth restricting effects of a single course of antenatal betamethasone treatment and the role of human placental lactogen. Placenta.

[23] Nugent BM, Bale TL (2015). The omniscient placenta: Metabolic and epigenetic regulation of fetal programming. Front Neuroendocrinol.

[24] Bronson SL, Bale TL (2016). The placenta as a mediator of stress effects on neurodevelopmental reprogramming. Neuropsychopharmacology.

[25] Myatt L (2006). Placental adaptive responses and fetal programming. J Physiol.

[26] Broad KD, Keverne EB (2011). Placental protection of the fetal brain during short-term food deprivation. Proc Natl Acad Sci USA.

[27] Wiechmann T, Roh S, Sauer S, Czamara D, Arloth J, Kodel M (2019). Identification of dynamic glucocorticoid-induced methylation changes at the FKBP5 locus. Clin Epigenet.

[28] Klengel T, Mehta D, Anacker C, Rex-Haffner M, Pruessner JC, Pariante CM (2013). Allele-specific FKBP5 DNA demethylation mediates gene-childhood trauma interactions. Nat Neurosci.

[29] Provencal N, Arloth J, Cattaneo A, Anacker C, Cattane N, Wiechmann T (2020). Glucocorticoid exposure during hippocampal neurogenesis primes future stress response by inducing changes in DNA methylation. Proc Natl Acad Sci USA.

[30] Braun F, Hardt AK, Ehrlich L, Sloboda DM, Challis JRG, Plagemann A (2018). Sex-specific and lasting effects of a single course of antenatal betamethasone treatment on human placental 11beta-HSD2. Placenta.

[31] Mansell G, Gorrie-Stone TJ, Bao Y, Kumari M, Schalkwyk LS, Mill J (2019). Guidance for DNA methylation studies: statistical insights from the Illumina EPIC array. BMC Genom.

[32] Zannas AS, Wiechmann T, Gassen NC, Binder EB (2016). Gene-stress-epigenetic regulation of FKBP5: clinical and translational implications. Neuropsychopharmacology.

[33] Fries GR, Gassen NC, Rein T (2017). The FKBP51 glucocorticoid receptor co-chaperone: regulation, function, and implications in health and disease. Int J Mol Sci.

[34] Roeh S, Wiechmann T, Sauer S, Kodel M, Binder EB, Provencal N (2018). HAM-TBS: high-accuracy methylation measurements via targeted bisulfite sequencing. Epigenet Chromatin.

[35] Paquette AG, Lester BM, Koestler DC, Lesseur C, Armstrong DA, Marsit CJ (2014). Placental FKBP5 genetic and epigenetic variation is associated with infant neurobehavioral outcomes in the RICHS cohort. PLoS ONE.

[36] Briffa JF, Hosseini SS, Tran M, Moritz KM, Cuffe JSM, Wlodek ME (2017). Maternal growth restriction and stress exposure in rats differentially alters expression of components of the placental glucocorticoid barrier and nutrient transporters. Placenta.

[37] Capron LE, Ramchandani PG, Glover V (2018). Maternal prenatal stress and placental gene expression of NR3C1 and HSD11B2: the effects of maternal ethnicity. Psychoneuroendocrinology.

[38] Chen HJ, Antonson AM, Rajasekera TA, Patterson JM, Bailey MT, Gur TL (2020). Prenatal stress causes intrauterine inflammation and serotonergic dysfunction, and long-term behavioral deficits through microbe- and CCL2-dependent mechanisms. Transl Psychiatry.

[39] Lian S, Guo J, Wang L, Li W, Wang J, Ji H (2017). Impact of prenatal cold stress on placental physiology, inflammatory response, and apoptosis in rats. Oncotarget.

[40] Litzky JF, Deyssenroth MA, Everson TM, Lester BM, Lambertini L, Chen J (2018). Prenatal exposure to maternal depression and anxiety on imprinted gene expression in placenta and infant neurodevelopment and growth. Pediatr Res.

[41] van Uitert M, Moerland PD, Enquobahrie DA, Laivuori H, van der Post JA, Ris-Stalpers C (2015). Meta-analysis of placental transcriptome data identifies a novel molecular pathway related to preeclampsia. PLoS ONE.

[42] Mansell T, Novakovic B, Meyer B, Rzehak P, Vuillermin P, Ponsonby AL (2016). The effects of maternal anxiety during pregnancy on IGF2/H19 methylation in cord blood. Transl Psychiatry.

[43] American College of Obstetricians and Gynecologists’ Committee on Obstetric Practice. Committee opinion no. 713 summary: antenatal corticosteroid therapy for fetal maturation. Obstet Gynecol. 2017;130(2):493–4. 10.1097/AOG.0000000000002231 28742672

[44] Jobe AH, Milad MA, Peppard T, Jusko WJ (2020). Pharmacokinetics and pharmacodynamics of intramuscular and oral betamethasone and dexamethasone in reproductive age women in India. Clin Transl Sci.

[45] Monk C, Feng T, Lee S, Krupska I, Champagne FA, Tycko B (2016). Distress during pregnancy: epigenetic regulation of placenta glucocorticoid-related genes and fetal neurobehavior. Am J Psychiatry.

[46] Kress C, Thomassin H, Grange T (2006). Active cytosine demethylation triggered by a nuclear receptor involves DNA strand breaks. Proc Natl Acad Sci USA.

[47] Rein T (2016). FK506 binding protein 51 integrates pathways of adaptation: FKBP51 shapes the reactivity to environmental change. BioEssays.

[48] Clayton DF, Anreiter I, Aristizabal M, Frankland PW, Binder EB, Citri A (2020). The role of the genome in experience-dependent plasticity: Extending the analogy of the genomic action potential. Proc Natl Acad Sci USA.

[49] Lagadari M, De Leo SA, Camisay MF, Galigniana MD, Erlejman AG (2016). Regulation of NF-kappaB signalling cascade by immunophilins. Curr Mol Pharmacol.

[50] Zannas AS, Jia M, Hafner K, Baumert J, Wiechmann T, Pape JC (2019). Epigenetic upregulation of FKBP5 by aging and stress contributes to NF-kappaB-driven inflammation and cardiovascular risk. Proc Natl Acad Sci USA.

[51] Han VX, Patel S, Jones HF, Nielsen TC, Mohammad SS, Hofer MJ (2021). Maternal acute and chronic inflammation in pregnancy is associated with common neurodevelopmental disorders: a systematic review. Transl Psychiatry.

[52] Girchenko P, Lahti-Pulkkinen M, Heinonen K, Reynolds RM, Laivuori H, Lipsanen J, et al. Persistently high levels of maternal antenatal inflammation are associated with and mediate the effect of prenatal environmental adversities on neurodevelopmental delay in the offspring. Biol Psychiatry. 2020;87(10):898–907. 10.1016/j.biopsych.2019.12.004 31987493

[53] Burton GJ, Sebire NJ, Myatt L, Tannetta D, Wang YL, Sadovsky Y (2014). Optimising sample collection for placental research. Placenta.

[54] Rudolph MD, Graham AM, Feczko E, Miranda-Dominguez O, Rasmussen JM, Nardos R (2018). Maternal IL-6 during pregnancy can be estimated from newborn brain connectivity and predicts future working memory in offspring. Nat Neurosci.

[55] Hantsoo L, Kornfield S, Anguera MC, Epperson CN (2019). Inflammation: a proposed intermediary between maternal stress and offspring neuropsychiatric risk. Biol Psychiatry.

[56] Hadlock FP, Harrist RB, Sharman RS, Deter RL, Park SK (1985). Estimation of fetal weight with the use of head, body, and femur measurements—a prospective study. Am J Obstet Gynecol.

[57] Mayhew TM (2008). Taking tissue samples from the placenta: an illustration of principles and strategies. Placenta.

[58] Chang CC, Chow CC, Tellier LC, Vattikuti S, Purcell SM, Lee JJ (2015). Second-generation PLINK: rising to the challenge of larger and richer datasets. Gigascience.

[59] Aryee MJ, Jaffe AE, Corrada-Bravo H, Ladd-Acosta C, Feinberg AP, Hansen KD (2014). Minfi: a flexible and comprehensive Bioconductor package for the analysis of Infinium DNA methylation microarrays. Bioinformatics.

[60] Maksimovic J, Gordon L, Oshlack A (2012). SWAN: Subset-quantile within array normalization for illumina infinium HumanMethylation450 BeadChips. Genome Biol.

[61] Leek JT, Johnson WE, Parker HS, Jaffe AE, Storey JD (2012). The sva package for removing batch effects and other unwanted variation in high-throughput experiments. Bioinformatics.

[62] Chen YA, Lemire M, Choufani S, Butcher DT, Grafodatskaya D, Zanke BW (2013). Discovery of cross-reactive probes and polymorphic CpGs in the Illumina Infinium HumanMethylation450 microarray. Epigenetics.

[63] Price ME, Cotton AM, Lam LL, Farre P, Emberly E, Brown CJ (2013). Additional annotation enhances potential for biologically-relevant analysis of the Illumina Infinium HumanMethylation450 BeadChip array. Epigenet Chromatin.

[64] McCartney DL, Walker RM, Morris SW, McIntosh AM, Porteous DJ, Evans KL (2016). Identification of polymorphic and off-target probe binding sites on the Illumina Infinium MethylationEPIC BeadChip. Genom Data.

[65] Westra HJ, Jansen RC, Fehrmann RS, te Meerman GJ, van Heel D, Wijmenga C (2011). MixupMapper: correcting sample mix-ups in genome-wide datasets increases power to detect small genetic effects. Bioinformatics.

[66] Hahne F, Ivanek R (2016). Visualizing genomic data using Gviz and bioconductor. Methods Mol Biol.

[67] Teschendorff AE, Breeze CE, Zheng SC, Beck S (2017). A comparison of reference-based algorithms for correcting cell-type heterogeneity in Epigenome-Wide Association Studies. BMC Bioinform.

[68] Yuan V, Hui D, Yin Y, Penaherrera MS, Beristain AG, Robinson WP (2021). Cell-specific characterization of the placental methylome. BMC Genom.

[69] Barfield RT, Kilaru V, Smith AK, Conneely KN (2012). CpGassoc: an R function for analysis of DNA methylation microarray data. Bioinformatics.

[70] Pidsley R, Zotenko E, Peters TJ, Lawrence MG, Risbridger GP, Molloy P (2016). Critical evaluation of the Illumina MethylationEPIC BeadChip microarray for whole-genome DNA methylation profiling. Genome Biol.

[71] Dieckmann L, Lahti-Pulkkinen M, Kvist T, Lahti J, DeWitt PE, Cruceanu C (2021). Characteristics of epigenetic aging across gestational and perinatal tissues. Clin Epigenet.

[72] Dobin A, Davis CA, Schlesinger F, Drenkow J, Zaleski C, Jha S (2013). STAR: ultrafast universal RNA-seq aligner. Bioinformatics.

[73] Liao Y, Smyth GK, Shi W (2014). featureCounts: an efficient general purpose program for assigning sequence reads to genomic features. Bioinformatics.

[74] Watanabe K, Taskesen E, van Bochoven A, Posthuma D (2017). Functional mapping and annotation of genetic associations with FUMA. Nat Commun.

