# Supplementary material for: Betamethasone administration during pregnancy is associated with placental epigenetic changes with implications for inflammation
Source: Clin Epigenetics. 2021 Aug 26;13:165. doi: 10.1186/s13148-021-01153-y (PMC8393766; doi:10.1186/s13148-021-01153-y)
Supplement: Supplementary file 1 — Additional file 1: Table S1. EPIC CpGs significantly associated with BET [file 13148_2021_1153_MOESM1_ESM.docx]

**Table S1:** FDR significant CpGs associated with BET

| **CpG** | **chr** | **bp** | **nominal p-value** | **gene** |
| --- | --- | --- | --- | --- |
| cg04314723 | 6 | 25,282,718 | 9,13x10^-08^ | *LRRC16A* |
| cg22363520 | 6 | 35,558,488 | 4,04x10^-11^ | *FKBP5* |

| CpG: CpG name |
| --- |
| chr: chromosome |
| bp: base-pair position (hg19) |
| nominal p-value: nominal p-value for association with BET |
| gene: gene the CpG is located in (based on Illumina annotation) |
