# Supplementary material for: Betamethasone administration during pregnancy is associated with placental epigenetic changes with implications for inflammation
Source: Clin Epigenetics. 2021 Aug 26;13:165. doi: 10.1186/s13148-021-01153-y (PMC8393766; doi:10.1186/s13148-021-01153-y)
Supplement: Supplementary file 2 — Additional file 2. Supplementary EWAS on child’s sex and gestational age [file 13148_2021_1153_MOESM2_ESM.docx]

***Placental DNA methylation (DNAm) is associated with sex and gestational age***

To be able to compare effect sizes of BET exposure to those of other predictors, we checked for association between DNA methylation (DNAm) levels and factors previously reported to be associated with placental DNAm: child’s sex (1) and gestational age at birth (2). We found 2,234 CpG sites differently methylated based on child’s sex at a false-discovery rate (FDR) of 0.05 (see Fig. S1 in Additional File 3 and Table S2 in Additional File 4). The mean absolute methylation difference in these associated hits was 3.5% (SD=0.02) between male and female offspring. There were 59 CpGs significantly associated with gestational age at FDR of 0.05 (see see Fig. S1 in Additional File 3 and Table S3 in Additional File 5). The mean absolute methylation difference was 2.3% (SD=0.01, based on comparison of individuals with gestational age below the median, i.e., 268 days of gestation, to individuals above the median).

Bibliography

1. Gong S, Johnson MD, Dopierala J, Gaccioli F, Sovio U, Constancia M, et al. Genome-wide oxidative bisulfite sequencing identifies sex-specific methylation differences in the human placenta. Epigenetics. 2018;13(3):228-39.

2. Novakovic B, Yuen RK, Gordon L, Penaherrera MS, Sharkey A, Moffett A, et al. Evidence for widespread changes in promoter methylation profile in human placenta in response to increasing gestational age and environmental/stochastic factors. BMC Genomics. 2011;12:529.
