# Supplementary figures and images for: Betamethasone administration during pregnancy is associated with placental epigenetic changes with implications for inflammation
Source: Clin Epigenetics. 2021 Aug 26;13:165. doi: 10.1186/s13148-021-01153-y (PMC8393766; doi:10.1186/s13148-021-01153-y)

A

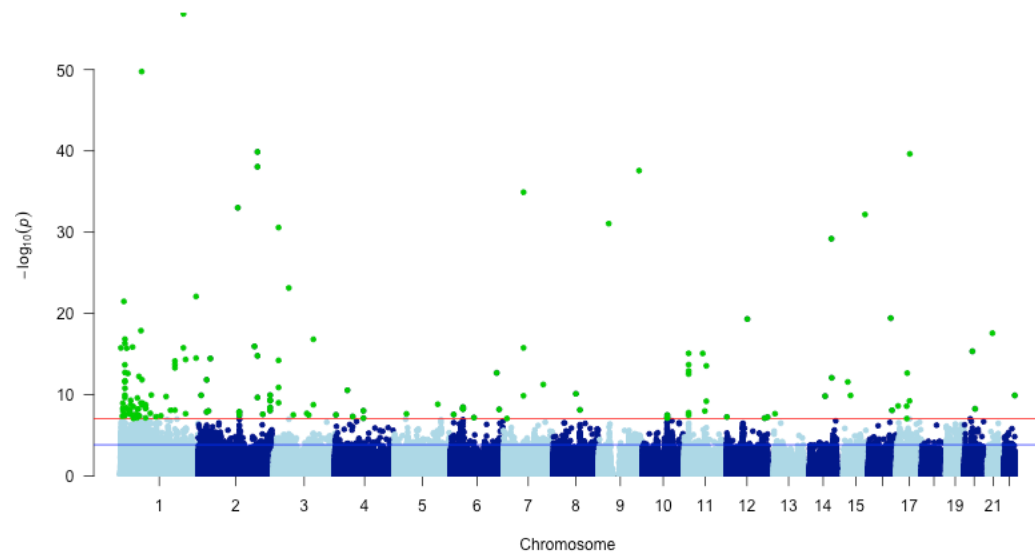

B

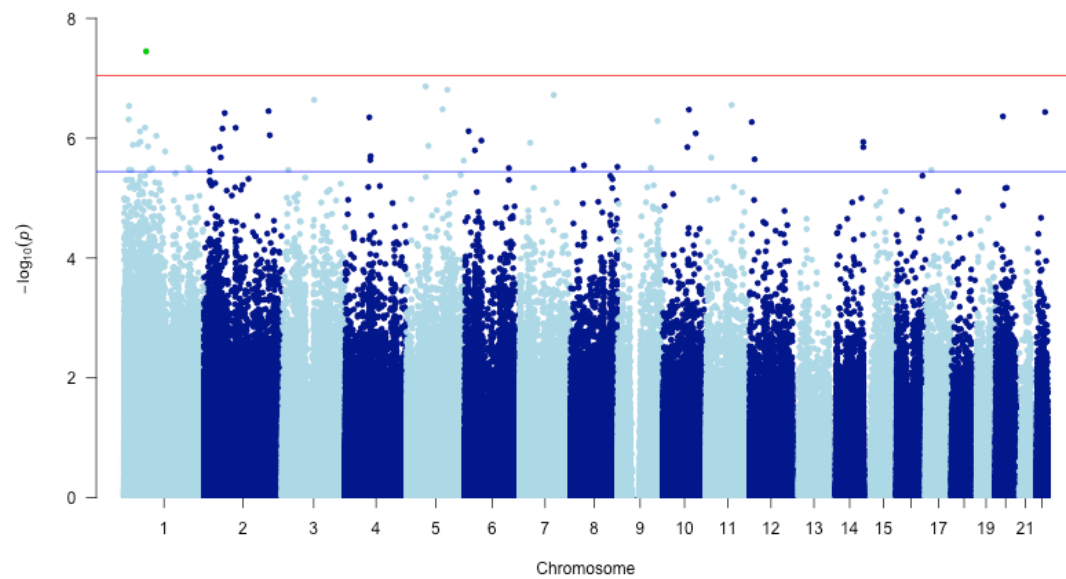

Figure S1

Supplement: Supplementary file 3 — Additional file 3: Figure S1. Association of DNAm levels with child’s sex and gestational age. Manhattan plot for association of child’s sex with DNA-methylation level. The position of the CpG site is depicted on the x-axis, the –log10(p value) on the y-axis. The red line indicates epigenome-wide significance, the blue line significance at FDR of 0.05 (A). Manhattan plot for association of gestational age with DNA-methylation level. The position of the CpG-site is depicted on the x-axis, the –log10(p value) on the y-axis. The red line indicates epigenome-wide significance, the blue line significance at FDR of 0.05 (B) [file 13148_2021_1153_MOESM3_ESM.pdf]

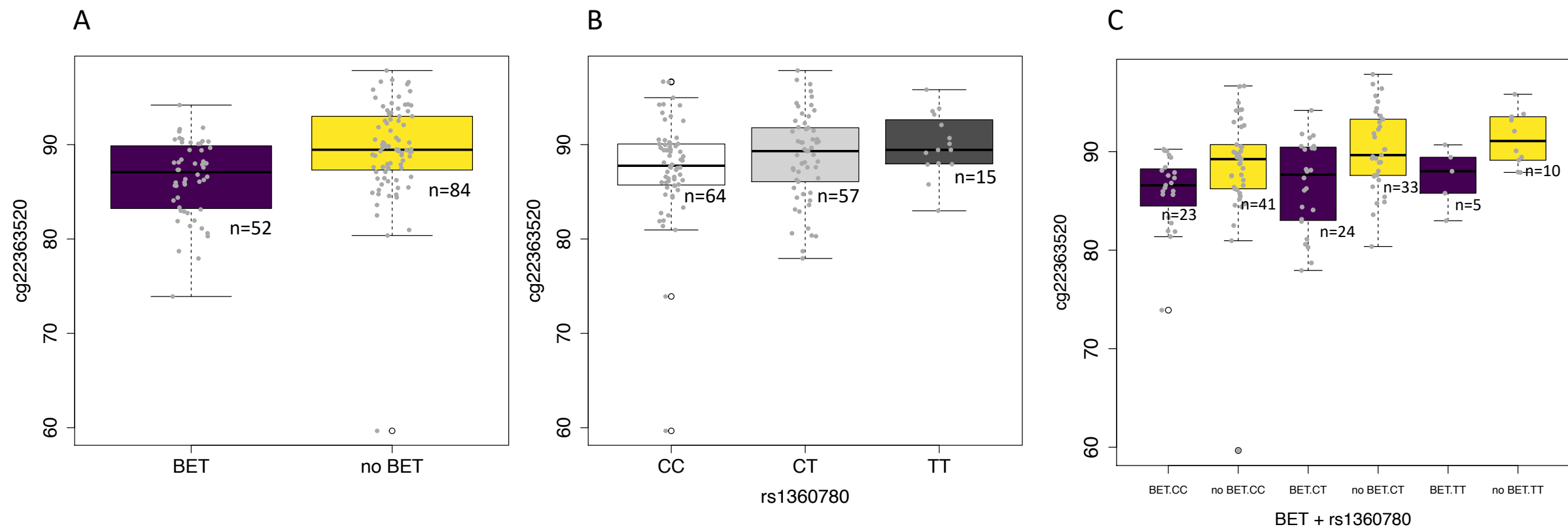

Figure S2

Supplement: Supplementary file 7 — Additional file 7: Figure S2. Boxplot of placental DNAm at cg22363520 and BET + SNP genotype in the TBS analysis. Boxplot of placental DNAm at cg22363520 and BET. The y-axis denotes placental DNAm of cg22363520, the x-axis denotes the betamethasone exposure group, depicted in purple, and the control group, depicted in yellow (A). Boxplot of placental DNAm at cg22363520 and rs1360780 genotype. The y-axis denotes placental DNAm of cg22363520, the x-axis denotes rs1360780 genotype (B). Boxplot of placental DNAm at cg22363520 and BET+ rs1360780 genotype. The y-axis denotes denotes placental DNAm of cg22363520, the x-axis denotes rs1360780 genotype and BET, e.g. ‘BET.CC‘ indicates the group presenting with BET and a CC genotype while ‘no BET.CT‘ indicates the group with no BET exposure and the CT genotype (C). [file 13148_2021_1153_MOESM7_ESM.pdf]
