# Supplementary material for: Betamethasone administration during pregnancy is associated with placental epigenetic changes with implications for inflammation
Source: Clin Epigenetics. 2021 Aug 26;13:165. doi: 10.1186/s13148-021-01153-y (PMC8393766; doi:10.1186/s13148-021-01153-y)
Supplement: Supplementary file 9 — Additional file 9: Table S6. Genes in the turquoise WGCNA module associated with prenatal stress [file 13148_2021_1153_MOESM9_ESM.docx]

**Table S6:** Genes in the turquoise WGCNA module associated with prenatal stress

| **genes** | **publication** | **type of stress** | **species** |
| --- | --- | --- | --- |
| *NR3C1* | Capron et al. | depression/anxiety/stressful life events | human |
| *AXL, CYR61,*  *MEG3, NDN* | Litzkey et al. | depression/anxiety | human |
| *CCL2* | Chen et al. | restraint stress | mice/rats |
| *SLC2A3* | Briffa et al. | physiological stress | mice/rats |
| *TLR4* | Lian et al. | cold stress | mice/rats |
