# Supplementary material for: Betamethasone administration during pregnancy is associated with placental epigenetic changes with implications for inflammation
Source: Clin Epigenetics. 2021 Aug 26;13:165. doi: 10.1186/s13148-021-01153-y (PMC8393766; doi:10.1186/s13148-021-01153-y)
Supplement: Supplementary file 13 — Additional file 13. Supplementary Methods [file 13148_2021_1153_MOESM13_ESM.docx]

## *Targeted bisulfite sequencing of the FKBP5 locus in the BET cohort*

This method has been previously described in detail in (1).

*Amplicon selection and amplification by PCR:* We optimized the amplifications of 19 regions covering 170 CpGs within glucocorticoid-receptor binding sites, as well as the transcription start site of the *FKBP5* locus. In order to reduce cost and maximize the number of samples per sequencing run, triplicate bisulfite treatments were performed for each sample and then pooled to run one PCR amplification per amplicon (1). Overall, 200 ng to 500 ng of DNA was used per sample and bisulfite treated using the EZ DNA Methylation Kit (Zymo Research, Irvine, CA). From this, 20 ng of bisulfite converted DNA was then used for each PCR amplification employing Takara EpiTaq HS Polymerase (Clontech, Saint-Germain-en-Laye, France) and 49 amplification cycles. PCR amplicons were then quantified with the Agilent 2200 TapeStation (Agilent Technologies, Waldbronn, Germany) and pooled in equimolar quantities for each sample. AMPure XP beads (Beckman Coulter, Krefeld, Germany) were used for a double size selection (200-500 bp) to remove primer dimers and high molecular DNA fragments.

*Sequencing*: Libraries were generated using the TruSeq DNA PCR-Free HT Library Prep Kit (Illumina, San Diego, CA) according to the manufacturer’s instructions. Each library was quantified with the Qubit® 1.0 (Thermo Fisher Scientific Inc., Schwerte, Germany), normalized to 4 nM and pooled. Library concentration and fragment sizes were checked via Agilent’s 2100 Bioanalyzer (Agilent Technologies, Waldbronn, Germany) and quantitative PCR using the Kapa HIFI Library quantification kit (Kapa Biosystems, Wilmington, MA). Paired-end sequencing was performed on an Illumina MiSeq Instrument (Illumina, San Diego, CA) with their MiSeq Reagent Kit v3 (2 x 300-cycles) with the addition of 30% of PhiX Library.

*Sequencing data processing:* The quality of the sequencing reads was checked with FastQC (http://www.bioinformatics.babraham.ac.uk/projects/fastqc) and Illumina adapter sequences were removed using Cutadapt (2). Bismark (https://www.bioinformatics.babraham.ac.uk/projects/bismark/) was used for the alignment to a restricted reference limited to our PCR targets. In order to stitch paired-end reads, an in-house Perl script has been developed to remove the low-quality ends of the paired-end reads if they overlapped. The methylation levels for all CpGs, CHGs and CHHs were quantified using the R package *methylKit* 1.8.1 (3). The resulting DNA methylation calls were submitted to a 3-step quality control. First, PCR artifacts introducing false CpGs of low coverage at 0 or 100% methylation level were removed. Second, CHH methylation levels were analyzed, and samples with insufficient bisulfite conversion rate (< 95%) were excluded. Finally, CpG sites with a coverage lower than 1,000 reads were removed. We excluded all CpGs with a callrate below 95% resulting in a final dataset of 106 CpG-sites. A list of all amplicons that passed quality control is given in Table S10 in Additional File 14. Three amplicons in PCR1 and PCR2 target exactly the same CpG-sites (PCR_1 at bp 127 equals PCR 2 at bp 29, PCR1 at bp 152 equals PCR 2 at bp 54 and PCR 1 at bp 205 equals PCR 2 at bp 107). All these amplicons passed quality control criteria and presented with association with the same effect direction in the statistical analyses. To remove redundancies, which can lead to inflated p-values, we used the 3 amplicons from PCR 2 in the final statistical analysis as these presented with higher coverage.

***EWAS on child’s sex and gestational age***

For EWAS on the child’s sex we used gestational age, the first four MDS components, pregnant person’s age, smoking during pregnancy, BET and the first two PCs from estimated cell type proportions as covariates. For EWAS on gestational age, child’s sex, the first four MDS components, pregnant person’s age, smoking during pregnancy, BET and the first two PCs from estimated cell type proportions were implemented.

***ITU cohort study population***

ITU study comprises two study arms. Individuals in the chromosomal testing arm (n=543) had been referred to the Helsinki and Uusimaa Hospital District Fetomaternal Medical Center (FMC) because they had an increased risk of fetal chromosomal abnormalities based on routine serum and ultrasound screening, age, and patient characteristics. They underwent fetal chromosomal testing (CVS, amniocentesis, or non-invasive prenatal testing) at FMC. If the chromosomal test indicated no fetal chromosomal abnormalities, those who had expressed interest in participating were contacted for final recruitment. Those whose chromosomal test results suggested a fetal chromosomal abnormality were not recruited. Individuals in the no chromosomal testing arm (n=401) were informed about ITU when attending the same routine serum and ultrasound screening at maternity clinics than the individuals in the chromosomal testing arm. Individuals who expressed interest in participating were contacted for final recruitment into this study arm if they had *not*been referred to FMC for fetal chromosomal testing. Both study arms provided placenta samples for this study.

***Weighted gene co-expression network analysis (WGCNA) in the ITU cohort***

We applied a variance stabilizing transformation to the raw counts of 8,245 transcripts from 494 individuals using the R package *DESeq2* 1.28.1 (4). Afterwards, a weighted gene co-expression network was constructed using the R package *WGCNA* 1.70-3 (5). Here, the absolute value of Pearson correlations between each pair of transcripts is calculated and transformed for a signed network (6). These similarity values are weighted by applying a soft-thresholding function, leading to the adjacency matrix. To compare pairs of genes in terms of their connection strength with all other genes in the network, a topological overlap measure is calculated (7). Following, a topology dissimilarity measure can be used as input for the hierarchical clustering procedure, to detect groups of genes (so called modules) that are highly co-expressed.

To find the appropriate soft-thresholding parameter, we used the function *pickSoftThreshold*, and the beta value with scale-free topology fit of R^2^ > .85. The according value was set as power parameter in the function *blockwiseModules* for the actual network construction and identification of modules. Further, the minimum module size was set to n = 30 genes, the threshold for merging modules was 0.25, and a signed network was chosen to preserve the sign of correlation between genes. To summarize, the overall expression of genes in a module, *eigengenes* were calculated by performing singular value decomposition of the module expression and extracting the first principal component.

***Expression quantitative methylation (eQTM) analysis***

As our top CpG from DNAm analysis was located in *FKBP5,* we focused on the gene module containing *FKBP5*, i.e., the turquoise module. We used variance stabilizing transformation on the raw gene expression counts of all genes in the turquoise WGCNA module*.* Afterwards, transformed expression levels were associated with beta-values of cg22363520, our top hit, using linear regression models and child’s sex, pregnant’s person age, smoking during pregnancy, gestational age as well as two first two principal components derived from estimated cell type proportions as described in (8) as covariates. Overall, 469 individuals had available information on cg22363520 and gene expression. P-values were corrected at a false-discovery rate (FDR)-cutoff of 5% using the Benjamini-Hochberg-approach over all genes included in the turquoise module.

Bibliography

1. Roeh S, Wiechmann T, Sauer S, Kodel M, Binder EB, Provencal N. HAM-TBS: high-accuracy methylation measurements via targeted bisulfite sequencing. Epigenetics Chromatin. 2018;11(1):39.

2. Marcel M. Cutadapt removes adapter sequences from high-throughput sequencing reads. EMBnet J. 2011;17:10-2.

3. Akalin A, Kormaksson M, Li S, Garrett-Bakelman FE, Figueroa ME, Melnick A, et al. methylKit: a comprehensive R package for the analysis of genome-wide DNA methylation profiles. Genome Biol. 2012;13(10):R87.

4. Love MI, Huber W, Anders S. Moderated estimation of fold change and dispersion for RNA-seq data with DESeq2. Genome Biol. 2014;15(12):550.

5. Langfelder P, Horvath S. WGCNA: an R package for weighted correlation network analysis. BMC Bioinformatics. 2008;9:559.

6. Zhang B, Horvath S. A general framework for weighted gene co-expression network analysis. Stat Appl Genet Mol Biol. 2005;4:Article17.

7. Yip AM, Horvath S. Gene network interconnectedness and the generalized topological overlap measure. BMC Bioinformatics. 2007;8:22.

8. Yuan V, Hui D, Yin Y, Penaherrera MS, Beristain AG, Robinson WP. Cell-specific characterization of the placental methylome. BMC Genomics. 2021;22(1):6.
