# Supplementary material for: Betamethasone administration during pregnancy is associated with placental epigenetic changes with implications for inflammation
Source: Clin Epigenetics. 2021 Aug 26;13:165. doi: 10.1186/s13148-021-01153-y (PMC8393766; doi:10.1186/s13148-021-01153-y)
Supplement: Supplementary file 14 — Additional file 14: Table S10. Amplicons in TBS [file 13148_2021_1153_MOESM14_ESM.docx]

**Table S10:** Amplicons in TBS

| **CpG** | **PCR number** | **PCR Name** | **PCR Position (hg19)** | **Quality control** |
| --- | --- | --- | --- | --- |
| intronic GRE intron 7 | 1 | FKBP5 int7 PCR 1.2 | chr6:35558361-35558652 | passed |
| intronic GRE intron 7 | 2 | FKBP5 int7 PCR 1.4 | chr6:35558459-35558774 | passed |
| intronic GRE intron 5 | 3 | FKBP5 int5 PCR 2 | chr6:35569680-35569946 | passed |
| intronic GRE intron 5 | 24 | FKBP5cg14284211 | chr6:35570168-35570410 | passed |
| intronic GRE intron 5 | 4 | FKBP5 int5 PCR 3 | chr6:35578686-35578916 | passed |
| intronic GRE intron 2 | 5 | FKBP5 int2 PCR 4 | chr6:35607754-35608065 | passed |
| intronic GRE intron 1 | 12 | FKBP5 PCR 11 | chr6:35630949-35631205 | passed |
| proximal enhancer | 13 | FKBP5 PCR 12 | chr6:35683267-35683538 | passed |
| proximal enhancer | 11 | FKBP5 promoter PCR 10 | chr6:35690247-35690512 | passed |
| proximal enhancer | 14 | FKBP5 PCR 13.1 | chr6:35693391-35693722 | failed due to coverage |
| proximal enhancer | 15 | FKBP5 PCR 13.2 | chr6:35693833-35694139 | passed |
| proximal enhancer | 16 | FKBP5 PCR 14 | chr6:35694529-35694831 | passed |
| proximal enhancer | 17 | FKBP5 PCR 15 | chr6:35695129-35695429 | passed |
| proximal enhancer | 18 | FKBP5 PCR 16.1 | chr6:35695657-35695960 | failed due to call rate |
| proximal enhancer | 19 | FKBP5 PCR 16.2 | chr6:35695938-35696263 | passed |
| proximal enhancer | 20 | FKBP5 PCR 17 | chr6:35696695-35697046 | passed |
| proximal enhancer | 21 | FKBP5 PCR 18 | chr6:35697684-35697842 | passed |
| proximal enhancer | 22 | FKBP5 PCR 19 | chr6:35699145-35699475 | passed |
| proximal enhancer | 24 | FKBP5cg14284211 | chr6:35570168-35570410 | passed |
| proximal enhancer | 29 | CTCF PCR 6 | chr6:35699693-35700085 | failed due to call rate |
