# Supplementary material for: Betamethasone administration during pregnancy is associated with placental epigenetic changes with implications for inflammation
Source: Clin Epigenetics. 2021 Aug 26;13:165. doi: 10.1186/s13148-021-01153-y (PMC8393766; doi:10.1186/s13148-021-01153-y)
Supplement: Supplementary file 15 — Additional file 15: Table S11. Estimated cell type proportions in whole sample, BET and controls [file 13148_2021_1153_MOESM15_ESM.docx]

**Table S11:** Estimated cell type proportions in whole sample. BET and controls

| **celltype** | **mean whole sample (SD). n=136** | **mean BET (SD) n=52** | **mean controls (SD) n=84** | **p-value** |
| --- | --- | --- | --- | --- |
| endothelial cells | 0.0876 (0.0288) | 0.0858 (0.0272) | 0.0887 (0.0298) | 0.5410 |
| Hofbauer cells | 0.0152 (0.0144) | 0.0171 (0.0154) | 0.0140 (0.0139) | 0.1365 |
| nRBC | 0.0024 (0.0086) | 0.0022 (0.0112) | 0.0025 (0.0066) | 0.0592 |
| stromal cells | 0.0972 (0.0291) | 0.1028 (0.0275) | 0.0937 (0.0296) | **0.0355** |
| syncytiotrophoblast | 0.6539 (0.0980) | 0.6553 (0.1095) | 0.6530 (0.0924) | 0.7051 |
| trophoblasts | 0.1437 (0.0743) | 0.1367 (0.0822) | 0.1481 (0.0691) | 0.2526 |

| p-value: nominal p-value from Wilcoxon-test |
| --- |
| nominally significant p-values below 0.05 are depicted in bold |
